# Targeted association mapping demonstrating the complex molecular genetics of fatty acid formation in soybean

**DOI:** 10.1186/s12864-015-2049-4

**Published:** 2015-10-23

**Authors:** Ying-hui Li, Jochen C. Reif, Yan-song Ma, Hui-long Hong, Zhang-xiong Liu, Ru-zhen Chang, Li-juan Qiu

**Affiliations:** The National Key Facility for Crop Gene Resources and Genetic Improvement (NFCRI)/Key Lab of Germplasm Utilization (MOA), Institute of Crop Science, Chinese Academy of Agricultural Sciences, 100081, Beijing, China; Department of Breeding Research, Leibniz Institute of Plant Genetics and Crop Plant Research (IPK), Gatersleben, Germany; Soybean Research Institute, Heilongjiang Academy of Agricultural Sciences, 150086 Harbin, China

**Keywords:** Cultivated soybean, SNP, Fatty acid modifier, LD-mapping, Favorable alleles

## Abstract

**Background:**

The relative abundance of five dominant fatty acids (FAs) (palmitic, stearic, oleic, linoleic and linolenic acids) is a major factor determining seed quality in soybean.

**Methods:**

To clarify the currently poorly understood genetic architecture of FAs in soybean, targeted association analysis was conducted in 421 diverse accessions phenotyped in three environments and genotyped using 1536 pre-selected SNPs.

**Results:**

The population of 421 soybean accessions displayed significant genetic variation for each FA. Analysis of the molecular data revealed three subpopulations, which reflected a trend depending on latitude of cultivation. A total of 37 significant (*p* < 0.01) associations with FAs were identified by association mapping analysis. These associations were represented by 33 SNPs (occurring in 32 annotated genes); another four SNPs had a significant association with two different FAs due to pleiotropic interactions. The most significant associations were cross-verified by known genes/QTL or consistency across cultivation year and subpopulations.

**Conclusion:**

The detected marker-trait associations represent a first important step towards the implementation of molecular-marker-based selection of FA composition with the potential to substantially improve the seed quality of soybean with benefits for human health and for food processing.

**Electronic supplementary material:**

The online version of this article (doi:10.1186/s12864-015-2049-4) contains supplementary material, which is available to authorized users.

## Background

Cultivated soybean (*Glycine max* L. Merr) produces seeds with 15 to 25 % oil and is primarily grown as a major source of plant edible oil [1, 2]. The nutritional value, flavor and stability of soybean oil is determined by its five dominant fatty acids (FAs), including saturated palmitic (16:0) and stearic (18:0), monounsaturated oleic (18:1), and polyunsaturated linoleic (18:2) and alpha-linolenic (18:3). Reduction of saturated palmitic acid and increase of unsaturated FA concentrations in soybean oil is desirable to improve human cardiovascular health. ω-6 linoleic and ω-3 linolenic acids are essential to humans but cannot be produced by human metabolism and therefore must be obtained from the diet. However, the presence of high levels of polyunsaturated fatty acids (PUFAs), especially linolenic acid, increases autoxidation which causes off-flavor, and reduces the shelf life of soybean oil.

The inheritance of the five dominant FAs in soybean is controlled by major and minor genes [3]. Identifying molecular marker or quantitative trait loci (QTL) associated with FAs using marker-assisted selection (MAS) would facilitate the development of improved varieties to meet the widespread demand for healthier soybean oil. Linkage mapping is the traditional strategy for the identification of QTL using bi-parental mapping populations and has relatively high power and a low false positive rate. Several QTLs related to FAs have been reported [3–9] and a number of molecular markers associated with unique FAs were developed subsequently [10–12]. However, the utilization in breeding programs of QTL/molecular markers in the development of MAS or backcrossing for altering FAs has been limited due to low consistency across different genetic backgrounds resulting from the small fraction of the possible alleles sampled. Effectiveness is further restricted by the limited resolution and accuracy of these QTLs resulting from the low number of recombination events within bi-parental mapping populations, especially in genomic regions with high levels of linkage disequilibrium (LD). Therefore, it is necessary to clarify the molecular basis of natural variation and identify molecular markers associate with unique FAs in unrelated soybean germplasm with broad genetic diversity.

LD-based association mapping enables the identification of putative nucleotide polymorphisms responsible for phenotypic differences denoted as quantitative trait nucleotide(s), QTN, by searching for marker-trait associations. Association mapping has four main advantages: high mapping resolution, rich allele number, a reduction in time spent establishing mapping populations and greater utilization in MAS. Therefore, association mapping is increasingly used to dissect the genetic architecture of complex quantitative traits in soybean using universal SNP chips (i.e. Universal Soy Linkage Panel 1.0 with 1536 SNPs, SoySNP6k BeadChip with 5361 SNPs or SoySNP50K iSelect BeadChip with 52,041 SNPs) [13–18], genotyping by sequencing (GBS) [19–22] or re-sequencing [23], as complementary approaches for linkage mapping. Based on these analysis, a set of QTNs had been obtained which are significantly associated with maturity, plant height, seed weight, oil content, protein content, and resistance to soybean cyst nematode, sclerotinia stem rot, or white mold. The genetic basis of FA production, however, has not been fully elucidated using the association mapping approach in soybean.

To identify maximum genetic and phenotypic diversity of FAs, extant genetic resources from representative Chinese soybean core and applied collections [24], were genotyped using a 1536 SNP (mainly non-synonymous) chip and phenotyped in this study over three years. Subsequently, a genome-wide scan for significant markers was performed for further understanding of the genetic basis of differences in FAs and to enable the effective use of FA genetic resources. The results suggest that the association mapping approach is valid for detecting favorable alleles for FAs in soybean.

## Methods

### Plant materials

**Fig. 1:**
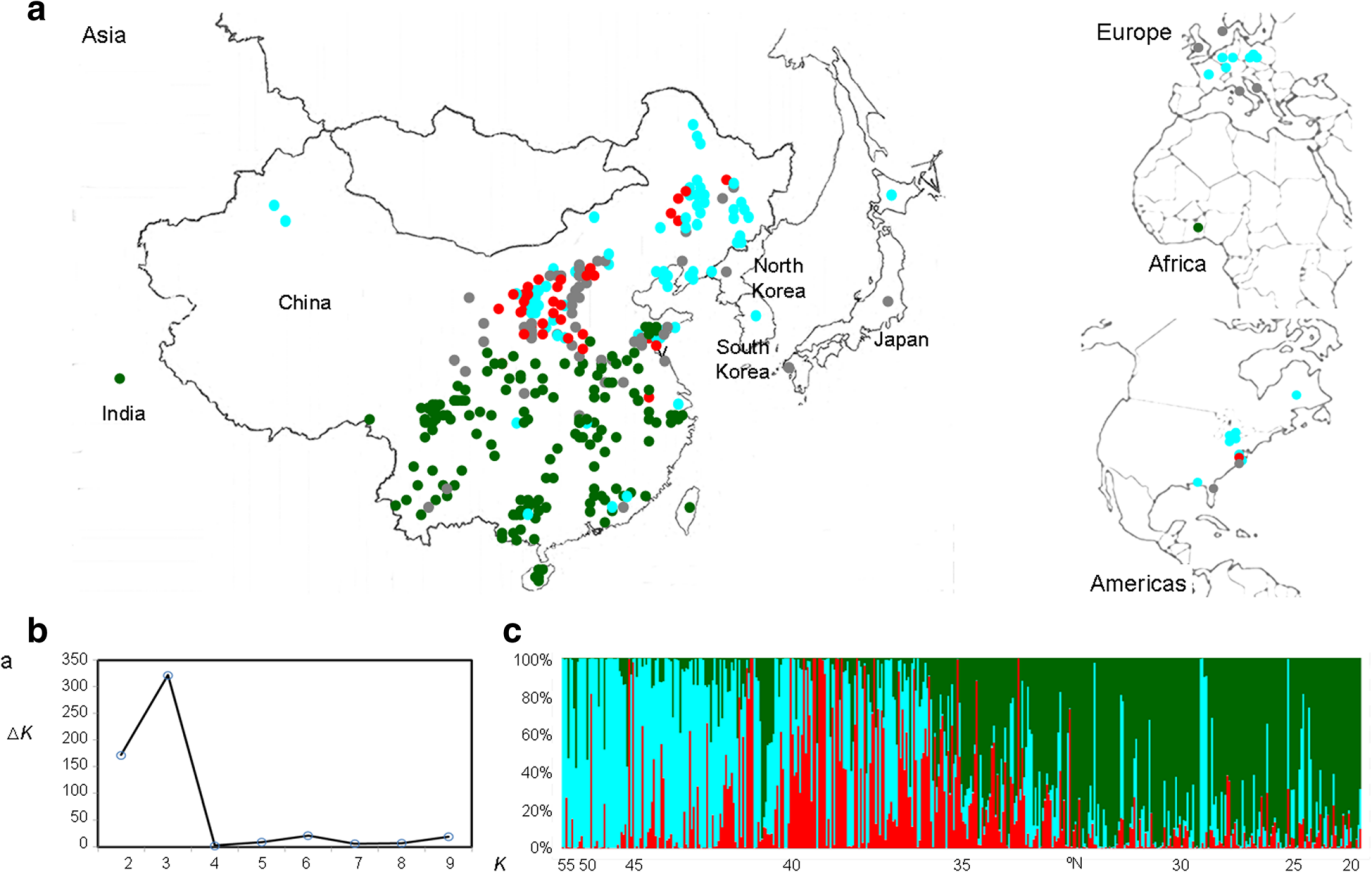
The geographic location, distributions and population structure across the range of cultivated soybean. **a** The geographic distributions of 421 soybean accessions analyzed in this study. The accessions assigned into three inferred genetic clusters (Northeast, central and South China) are indicated by solid blue, red and green circles respectively. Accessions with mixed genomes are indicated by the solid gray circle. **b** Inferred population structure of the soybean panel partitioned into three segments (K = 3). Each color represents one cluster, and the length of the colored segment shows the accession’s estimated proportion of membership in that cluster as calculated by STRUCTURE. The accessions are shown in order of latitude (°N) of their origin

A worldwide set of 421 soybean accessions was selected (Additional file 1) comprising 248 genotypes from the Chinese mini-core collection, 142 lines from the applied core collection of the Chinese National Soybean GeneBank (CNSGB) and 31 accessions from other countries worldwide (Fig. 1a). The lines from the Chinese mini-core and applied core collections have been described elsewhere [24, 25]. Each accession used in this study has been examined for phenotypic and/or genotypic homogeneity.

### Phenotyping data collection and analysis

The 421 accessions were evaluated in field trials in 2010, 2011 and 2012 in Sanya (SY10, SY11 and SY12, 18.2°N, 109.5°E), Hainan Province, China. The experiments were conducted following sowing in early January to early March each year. In all years, three biological replicates (rows) were planted following a complete random design. A row was 0.55 m wide and 1.5 m long with a spacing between plants of 0.10 m.

Seed was harvested after the accessions were grown to final maturity. The seeds from three replicate samples were pooled by accession and analyzed for FA components at the Agricultural Experiment Station chemical laboratories of the Chinese Agricultural University (CAU) in 2010, 2011 and 2012, respectively. The FA components were analyzed using an HP6890 gas chromatogram (GC) (Agilent Technologies, Palo Alto, CA, USA). Individual FA contents were calculated as percentage (%) of total free FA. All of the data were normalized by using both the seed weight and an internal reference. FA data used in the association mapping was the average of three replicates for a genotype. Variance component and heritability of target traits were analyzed using the R software lme4 package assuming random genotype and environment effects (http://www.r-project.org/). Pearson moment correlations between the five FAs pairwise were calculated by PASW statistics [26].

### SNP genotyping data collection

Twenty-four metabolic pathways and more than 600 annotated genes have been reported to be associated with the biosynthesis and degradation of acyl-lipids in *Arabidopsis* [27]. To dissect the genetic basis of FA production in soybean, using a targeted association mapping approach, 1794 putative homologous genes were annotated in the palaeopolyploid soybean by sequence comparison of known gene families in *Arabidopsis* [27, 28]. A total of 1536 single-nucleotide polymorphism (SNP) were selected from the group of SNPs produced by comparison of 55 whole-genome re-sequenced soybean genomes [29, 30] and the first soybean transcript map [31], to design a SNP genotyping array, which originated from putative homologous genes [28] and known QTL regions [5, 7, 8, 32, 33], randomly selected along chromosomes. Four hundred and twenty-one soybean accessions were assayed using the Illumina BeadArray platform (Illumina Inc., San Diego, CA, USA) following the manufacturer’s protocol. DNA was extracted from a bulk of young leaf tissue of 20–30 plants per accession as previous described [34]. A group of 242 SNPs with a GenCall score of < 80 % and GenTrain < 0.6 were excluded from further analysis since it was difficult to separate homozygote and heterozygote clusters, as described in our previous study [25]. A further 69 SNPs were removed due to either an excessive failure rate (10 % or more of samples) or to apparent heterozygosity (>20 % of samples). Finally, 20 polymorphic loci with minor allele frequency (MAF) < 5 % were removed from association mapping analysis. In the end, the final data set reflected the allelic state at 1205 SNP loci (Additional file 2)

### Analysis of population structure

Summary statistics, including the number of alleles, the frequency of major allele, gene diversity, the proportion of heterozygous individuals in the population and heterozygosity for each SNP locus were calculated using PowerMarker 3.25 software [35]. A subset of 756 SNPs which were evenly distributed across all 20 soybean chromosomes was selected to determine the population structure (Additional file 2), using Bayesian Markov Chain Monte Carlo approaches incorporated in the software package STRUCTURE 2.1 [36]. The admixture and independent allele frequency model was employed, using cluster number (*K*) ranging from 1 to 10. Twenty runs were performed for each value of *K*, without using previous population information, with a 100,000 burn-in length and 100,000 iterations. The estimated log likelihood values increased as the values of *K* increased, which indicated no clear genetic structure so the derivative of the log likelihood (∆*K*) was used to determine the most likely number of sub-clusters as previously reported [37].

### Association mapping

Marker/trait associations were tested for 1205 SNP loci with each of five averaged FAs using TASSEL 4.0 standalone software (http://www.maizegenetics.net). Since genome wide association studies can be susceptible to false positive associations from population stratification, a mixed linear model (MLM) method [38] with subpopulation membership percentage (*Q* matrix) and Kinship (*K* matrix) were performed. Q matrix and K matrix were inferred from the STRUCTURE and TASSEL 4.0 standalone programs, respectively, using allelic data from 756 evenly distributed SNP markers (Additional file 2). The threshold *p*-values for significant marker-traits associations were set at 0.001 by considering the scale of SNPs used in this study.

## Results and discussion

### Genotyping results

Four hundred and twenty-one soybean accessions were fingerprinted by a genotyping array designed with 1536 SNPs known to be present in 55 representative soybean accessions [29]. After exclusion of SNPs with a high failure rate or heterozygosity, 1205 markers met the threshold of quality control in this panel. Since an annotated gene approach was adopted to design the genotyping array, the majority (93 %) of the 1205 SNP were located in coding regions (CDS), the untranslated region (UTR) and the introns of 1074 annotated genes (Glyma v1.1). Of the 1077 SNPs in coding regions, 345 (32 %) were synonymous whereas 702 (65.2 %) were non-synonymous. In addition, 16 SNPs created a stop codon and 14 were found to have caused a change of open reading frame. The detailed information for each SNP can be found in Additional file 2.

### Population structure

For inferring population structure in the 421 soybean accessions, 756 SNPs evenly distributed across 20 soybean chromosomes were selected from 1205 SNPs. STRUCTURE analysis showed that the logarithm of the data likelihood (Ln P(D)) continued to increase with increasing numbers of assumed subpopulations (*K*) from 1 to 10. The ad-hoc quantity based on the second order rate of change in the log probability (Δ*K*) revealed that the uppermost model value of *K* was at *K* = 3 suggesting three genetically distinct subpopulations with limited evidence of admixture among them (Fig. 1b, c). The three subpopulations illustrated a trend related to latitude of original cultivation (Fig. 1a, c), and were subsequently denoted as NER (North East region of China), NR (North region of China) and SR (South region of China). The NER subpopulation consisted of 117 accessions mainly from high latitude areas (>40° N, 112 accessions); the NR subpopulation comprised 48 accessions mainly from areas between latitudes of 35–40° N (33 accessions); and SR subpopulation contained 180 accessions mainly from low latitude areas (<35° N, 172 accessions).

### Phenotyping and statistical analysis for five FA components

Four hundred and twenty-one diverse soybean accessions were evaluated in field trials across three years in Sanya, the southernmost city in China. Five dominant FAs of the seeds, i.e. linoleic, linolenic, oleic, palmitic and stearic acids, were determined using gas chromatography. Abundant variations were observed which ranged from a 1.2-fold difference in palmitic acid component to a 1.8-fold difference in linolenic acid (Table 1). Correspondingly, broad-sense heritability values for all of five FA components obtained from three years of phenotypic characterization were moderate to high with a range from 0.5 to 0.7.

**Table 1:** Phenotypic variation of five fatty acid components across soybean accessions

Traits	Mean	Min	Max	CV (%)	H^2^
Linoleic acid (%)	50.5	44.1	55.5	3.5	0.55
Linolenic acid (%)	9.1	6.7	12.1	8.8	0.70
Oleic acid (%)	25.5	20.0	31.6	7.6	0.51
Palmitic acid (%)	12.4	11.0	13.8	2.8	0.55
Stearic acid (%)	3.8	3.1	5.0	4.5	0.52

*H* ^*2*^ broad-sense heritability; *min* minimum value; *max* maximum value; *mean* mean values; *CV* coefficient of variation

**Fig. 2:**
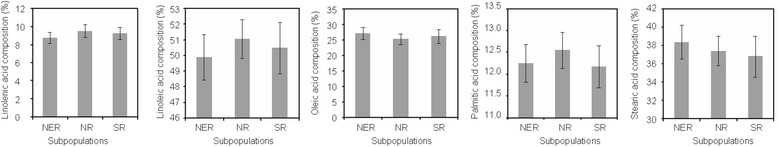
Variation in five fatty acids among different subpopulations of the soybean diversity panel. The error bar represents the standard error. NER, NR and SR contained 117, 48 and 180 soybean accessions, respectively

**Fig. 3:**
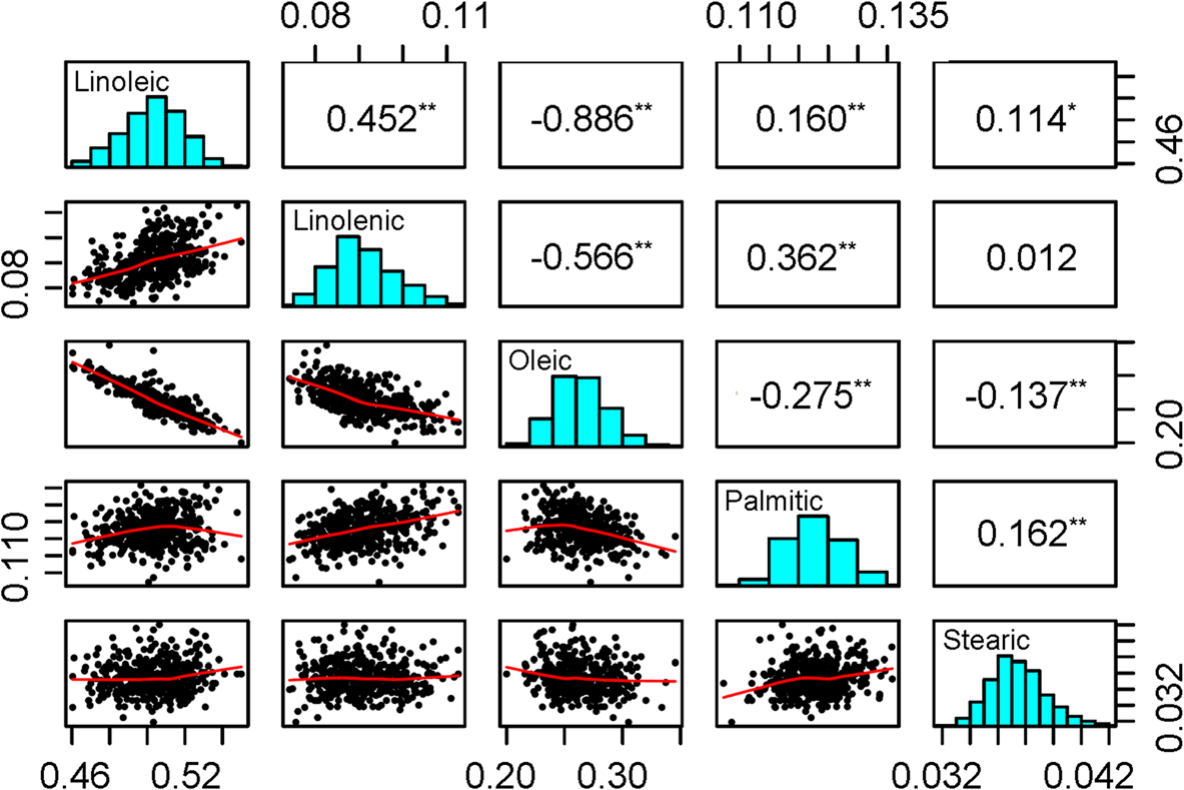
Pearson’s co-efficient of correlation for pairwise comparison of five fatty acids in soybean accessions. Differences significant at *p* < 0.05 are marked *; those significant at *p* < 0.01 are marked **

These five FAs showed significant differences among the three subpopulations (*p* at 0.05 level) (Fig. 2). NER had the lowest proportions of linolenic and linoleic acids, and the highest amounts of oleic and stearic acids. NR had the lowest oleic acid level and the highest linoleic and palmitic acid components. SR had the lowest levels for both palmitic and stearic acids. These differences may be attributed to natural selection due to environment factors such as temperature and planting days as well as artificial selection during domestication and genetic improvement. For example, the presence of a high component of linolenic acid is responsible for autoxidative instability and off-flavors associated with the oils, and thus farmers or breeders have always selected or developed soybean accessions with inherently low levels of linolenic acid. It has been reported that on average cultivated soybeans contain only around two-thirds of the linolenic acid found in wild soybean (*Glycine soja* Sieb. & Zucc., the progenitor of cultivated soybean) [39]. Wild soybean accessions produced up to 23 % linolenic acid [40, 41]. This study detected a significant difference in linolenic acid contents between the three subpopulations. Accessions from NER, the main high-oil soybean producing area in China, contained the lowest proportion of linolenic acid (8.7 %), followed by SR (9.3 %) and NR (9.5 %). These results agree with the suggestion that linolenic acid levels underwent selection to meet the demands of oil industry during domestication and the expansion of domesticated soybean production [39, 42]. The patterns of correlation coefficients (*r*) calculated across all five traits (Fig. 3) coincided with the previous observations [43]. Oleic acid levels were significantly negatively correlated (*p* < 0.01) with the other four traits. The closest relationship was detected with linoleic acid (*r* = −0.89) and the least significant relationship with stearic acid (*r* = −0.14). Most of the relationships among the other four traits were significantly (*p* < 0.01) positive. Among them, linolenic acid showed the closest relationship with linoleic acid (*r* = 0.45), followed by palmitic acid (*r* = 0.36). The relationship between linolenic and stearic acids was essentially random, with an *r* value of 0.01.

### Marker-trait association analysis

**Fig. 4:**
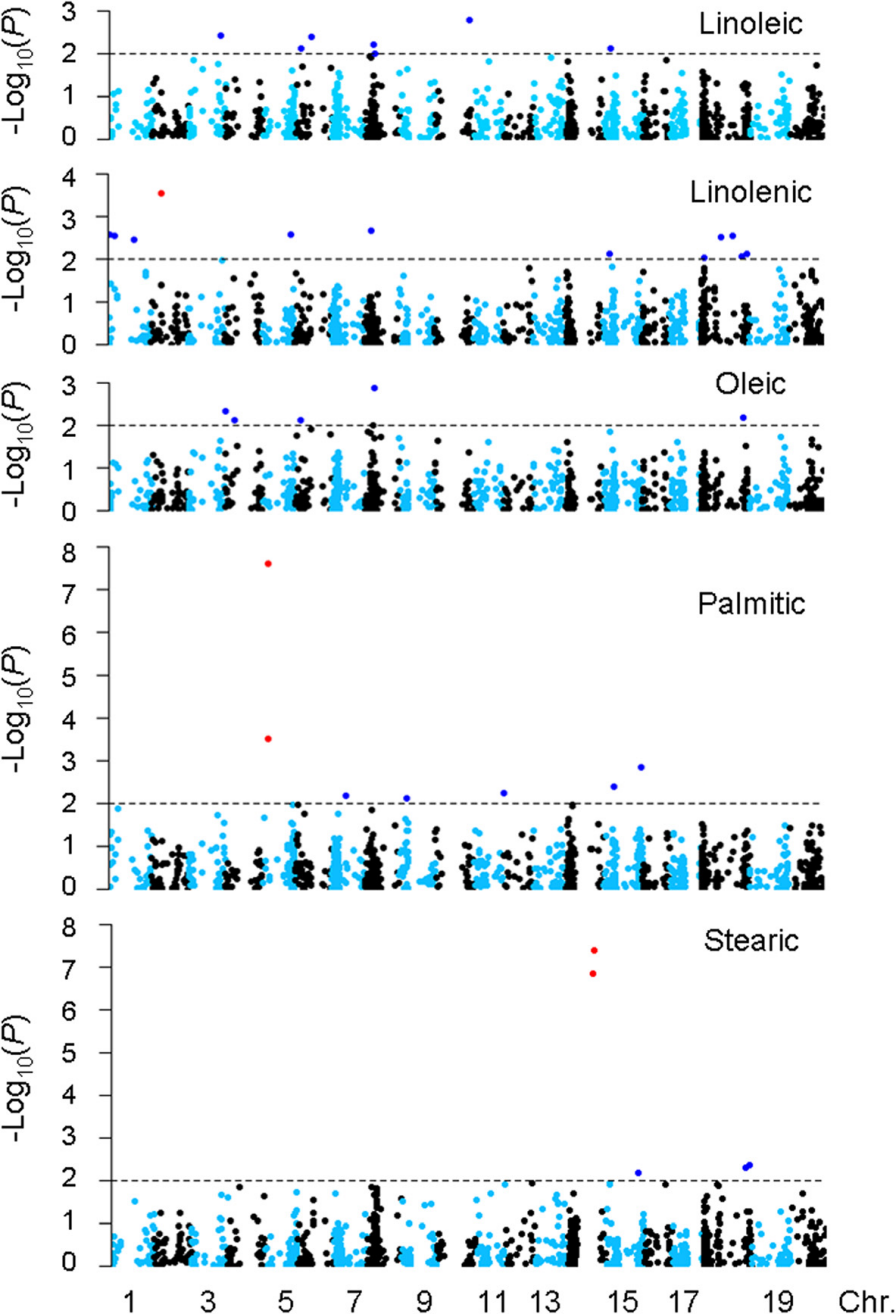
Manhattan plots of *p* values indicating SNP markers associated with five fatty acids. The x- axis shows SNP markers along each soybean chromosome; y- axis is the –log10 (*p* value), horizontal lines designate 1E-02 thresholds for significant associations. The association of SNP markers with highly significant associations (*p* < 0.001) are shown by red dots

Following the distinct population structure detected within this panel by STRUCTURE, the MLM model, which takes account of both kinship matrix and genetic structure (K + Q), was used to reveal QTNs associated with the five FAs. In total, 37 significant (*p* < 0.01) marker-trait associations were identified for these FA (Table 2). Around one-third of these QTNs were located within or near known QTLs (Table 2). The number of QTNs in the current study associated with a single FA ranged from five for oleic acid and stearic acid to 12 for linolenic acid (Fig. 4). A high consistency of associations (30 of 37 QTNs) was found between significant SNPs and alleles with the target trait in at least two of three environments. These QTNs were represented by 33 unique SNPs and lay in 32 annotated genes. Four SNPs (Map-3670, −6135, −6325, and −6520) exhibited significant (*p* < 0.01) associations with more than one trait. Of the 32 SNPs with significant (*p* < 0.01) associations, 28 were located in coding regions (87.5 %). The effects of 18 of these were determined: non-synonymous polymorphisms (15), stop codons (2) and frameshift (1) (Table 2). In the following we discuss in detail the marker-trait associations found for each of the five FAs:

**Table 2:** Summary of significant marker-trait associations identified by mixed model association mapping in the panel of 421 diverse soybean accessions

Trait	Marker name	Gene ID	*P* value	R^2^	Annotation/Pathway^a^	Marker/QTL reported in the previous studies		
						Marker/region	Physical position	Reference
Linoleic	BARC-028709-05992	Glyma03g32920	3.9E-03	0.020	Oxidoreductase activity	Sat_304_Satt022	41,100,977_44,682,615	[7]
	Map-6135	Glyma06g10830	7.6E-03	0.017	DNA photolyase activity			
	Map-6146	Glyma06g24830	4.1E-03	0.027	Molecular chaperone			
	Map-6283	Glyma08g14200	6.2E-03	0.018	Protein binding			
	Map-6325	Glyma08g17170	7.4E-03	0.017	Motor activity			
	Map-6326	Glyma08g17190	1.0E-02	0.022	Fatty acid elongation & Wax biosynthesis, Lipid transfer protein^a^			
	Map-6383	Glyma10g36250	1.7E-03	0.024	_	Satt153	45,959,176	[32]
	Map-6520	Glyma15g11810	7.5E-03	0.024	Malonyl-CoA decarboxylase^a^			
Linolenic	Map-0008	Glyma01g01830	2.7E-03	0.022	Catalytic activity			
	BARC-017909-02439	Glyma01g07120	2.8E-03	0.022	Ureide permease			
	Map-0076	Glyma01g24850	3.6E-03	0.028	Galactosyltransferases			
	Map-6017	Glyma02g15600	2.9E-04	0.032	SACPD-B or FAB2; Fatty acid synthesis and Fatty acid elongation^a^	The deletion of an ‘A’ nucleotide	14,107,987	[45]
	Map-6077	Glyma05g34030	2.6E-03	0.022	Hosphatidylethanolamine binding protein^a^			
	Map-6270	Glyma08g13060	2.1E-03	0.030	Protein kinase activity			
	Map-6520	Glyma15g11810	7.6E-03	0.024	Malonyl-CoA decarboxylase^a^	Satt384	4,036,564	[41]
						BARC-063195-18266_ BARC-028907-06042	5,085,811_ 5,522,353	[59]
	Map-6738	Glyma18g05980	9.4E-03	0.016	_			
	Map-3569		3.0E-03	0.022				
	Map-3580	Glyma18g34290	2.8E-03	0.029	_			
	Map-3665	Glyma18g43900	9.1E-03	0.023	Dirigent-like protein	Sat_164	53,656,448	[33]
	Map-6782	Glyma18g51400	7.5E-03	0.017				
Oleic	Map-6056	Glyma03g41850	4.7E-03	0.020	Phospholipid signaling^a^			
	BARC-042719-08393	Glyma04g12490	7.6E-03	0.017	Protein binding			
	Map-6135	Glyma06g10830	7.7E-03	0.017	DNA photolyase activity			
	Map-6325	Glyma08g17170	1.3E-03	0.025	Motor activity			
	Map-3670	Glyma18g44690	6.5E-03	0.018	MAC/Perforin domain	Sat_164	53,656,448	[33]
Palmitic	Map-0751	Glyma05g07630	3.1E-04	0.040	Strictosidine synthase activity	*GmFATB1a*	1,127,438_1,131,632	[60]
	Map-6064	Glyma05g07730	2.4E-08	0.077	Translocase^a^			
	Map-1323	Glyma07g18890	6.8E-03	0.025	Carbohydrate binding	Satt175	15,307,093	[61]
	Map-6356	Glyma09g09800	7.7E-03	0.024	PPR repeat	Satt544	11,309,091	[8]
	Map-6395	Glyma12g01380	5.8E-03	0.025	Triacylglycerol biosynthesis and Eukaryotic phospholipid synthesis & Editing^a^			
	Map-6540	Glyma15g17090	4.2E-03	0.020	Sequence-specific DNA binding transcription factor activity			
	Map-6590	Glyma15g41680	1.4E-03	0.032	Cell cycle control protein			
Stearic	BARC-013927-01275	Glyma14g27380	1.4E-07	0.078	Transcription cofactor activity	Sat_189	33,180,365	[32]
						Satt474	33,076,661	[49]
	Map-6506	Glyma14g27990	4.0E-08	0.085	SACPD-C or FAB2C; Fatty acid synthesis^a^	Sat_189	33,180,365	[32]
						Satt474	33,076,661	[49]
	Map-6568	Glyma15g37410	6.5E-03	0.018	Serine/Threonine protein kinase			
	Map-3670	Glyma18g44690	4.9E-03	0.019	MAC/Perforin domain	Satt288	55,407,034	[62]
	Map-6776	Glyma18g50790	4.4E-03	0.026	Iron ion binding	Sct_199_Sat_064	58,093,451_60,612,567	[8]

^a^Deduced metabolic pathways in soybean by sequence comparison of known gene families in *Arabidopsis* [27]

#### Linoleic acid

A total of eight significant marker-trait associations were detected each located in a distinct candidate gene associated with linoleic acid biosynthesis (Fig. 4, Table 2). Three SNPs were non-synonymous (Map-6283, −6325 and −6520). Individually, each of the eight putative QTNs explained a small proportion of the phenotypic variance, with effects ranging from 1.7 % (Map-6135 and −6325) to 2.7 % (Map-6146). Of eight QTNs, two (Map-6325 and −6326) were neighboring with a 41.4-kbp genomic distance. Map-6326 is a synonymous polymorphism in *Glyma08g17190*, which was predicted to be involved in fatty acid elongation and the wax biosynthesis pathway by comparison with homologs in *Arabidopsis thaliana* [28]. We identified a strong pairwise LD between Map-6325 and −6326 with r^2^ of 0.854.

#### Oleic acid

Five significant (*p* < 0.01) marker-trait associations were observed, which explained 1.7-2.5 % of oleic acid component variation (Table 2, Fig. 4). These SNPs lay in five annotated genes and two of them (Map-6325 and −3670) were deduced to be non-synonymous polymorphisms. Map-6056 was a synonymous polymorphism in *Glyma03g41850* which, by comparison with homologs in *Arabidopsis thaliana,* is likely to be involved in the phospholipid signaling pathway.

#### Linolenic acid

**Fig. 5:**
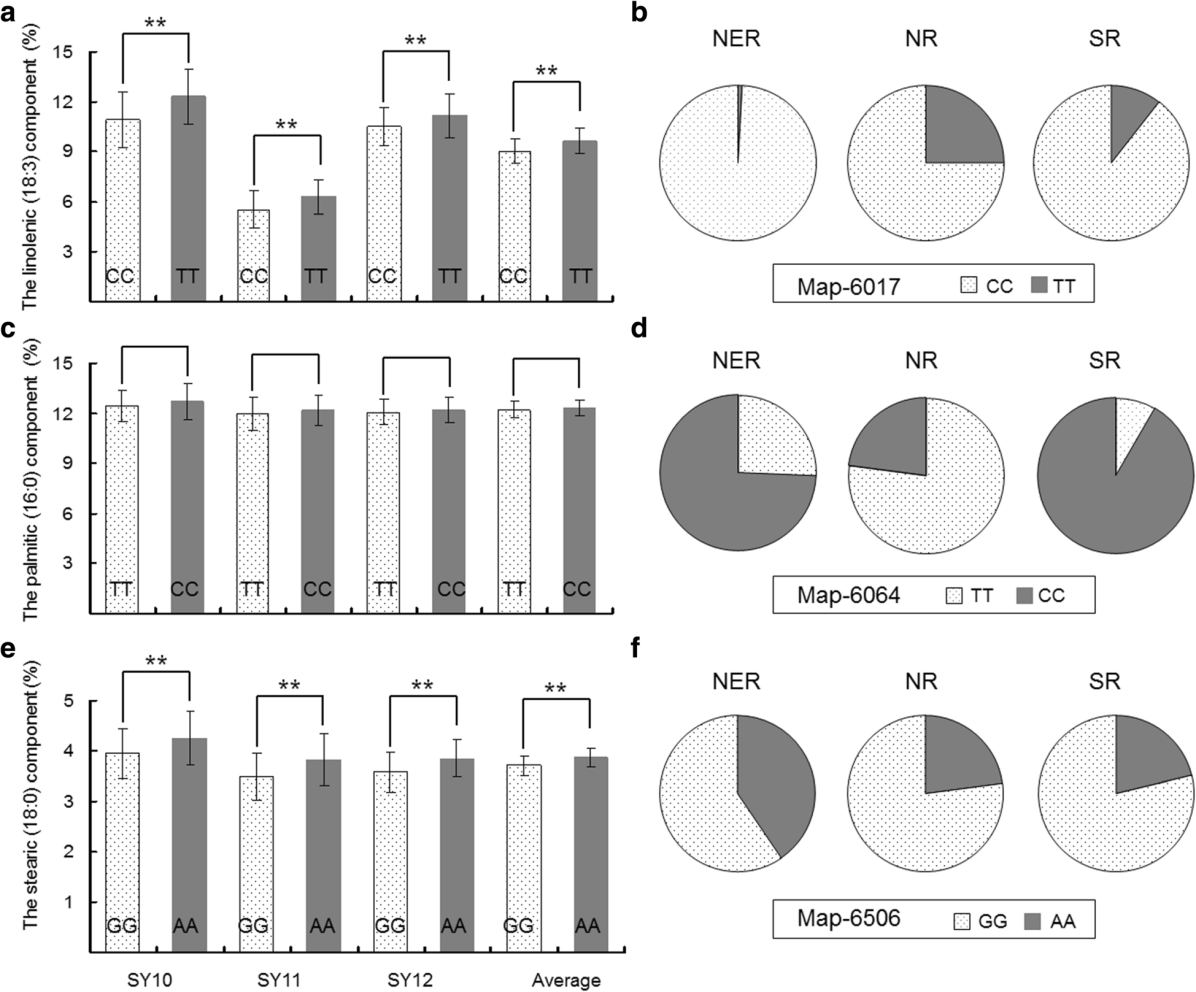
Diagrams depicting the genetic effects on associated fatty acids across three years, and allele frequencies in three subpopulations inferred from STRUCTURE analysis at three most significant SNP (*p* < 0.001) loci. **a** Linolenic acid bi-alleles at Map-6017. **b** Bi-allele frequency at Map-6017. **c** Palmitic acid bi-alleles at Map-6064. **d** Bi-allele frequency at Map-6064. **e** Stearic acid bi-alleles at Map-6506. **f** Bi-allele frequency at Map-6506. SY10, SY 11 and SY12 respectively indicate 2010, 2011 and 2012 growing seasons in Sanya

Twelve Significant (*p* < 0.01) marker-trait associations were detected located on Gm01 (3), Gm02 (1), Gm05 (1), Gm08 (1), Gm15 (1), and Gm18 (5). Map-6017, a synonymous polymorphism within *Glyma02g15600*, had the most significant association (*p* = 2.9E-04) and explained 3.2 % of the variation in proportion of linolenic acid. *Glyma02g15600* encoded one of three isoforms of △9-stearoyl-acyl carrier protein-desaturase (*SACPD-B* or *GmFAB2B*) [44, 45] and was predicted to play a role in fatty acid synthesis and fatty acid elongation [28]. It has been reported that a 1-bp insertion in exon 3 of *SACPD-B* which caused 28 amino acids changes compared to Williams 82 *SACPD-B*, was associated with linolenic acid production in soybean [45]. Map-6017 was 46-bp away from this 1-bp insertion. For each year, the accessions with allele T had significantly (*p* < 0.01) higher linolenic acid than those with allele C (Fig. 5a). Allele T in the SNP Map-6017 is a minor allele with a frequency of 9.5 % in this representative cultivated soybean panel, suggesting that this locus potentially underwent selection for low linolenic acid levels during soybean domestication. Further evidence that selection against allele T took place in genetic improvement arises from the decrease in frequency from landrace (13 %) to modern cultivars (2.2 %). Geographical distribution of bi-alleles in Map-6017 was uneven across the three subpopulations. The allele T occurred in 25 % of accessions from NR (the predicted domestication site of cultivated soybean [34]), its frequency decreased to 10.5 % in SR and to 0.9 % in NER subpopulations (Fig. 5b). These findings clearly suggest that this locus has been strongly selected in NER for production of linolenic acid levels.

#### Palmitic acid

Of seven SNPs associated with palmitic acid, Map-0751 and 6064 were the two most significant loci. They cover a 55-kbp genomic region. The most significant locus Map-6064 (*p* = 2.4E-08) explained 7.7 % of the phenotype variation and is a synonymous SNP within *Glyma05g07730*, a homolog of a potentially lipid trafficking gene in *Arabidopsis thaliana* (*AT3G25610*) [46]. In addition, Map-6064 is located 288-kbp upstream of *Glyma05g08060* (*GmFATB1a*). A nonsense mutation within exon 1 of *GmFATB1a* was previously found to be responsible for the reduction of palmitic acid in soybean [47]. Non-synonymous Map-0751 in *Glyma05g07630* was found to have significant LD (*p* =3.6E-44) with Map-6064 (r^2^ = 0.53, D’ = 0.98).

Since saturated palmitic acid give rise to negative health effects in humans associated with a diverse lipoprotein profile arising from consumption of this fatty acid [48], soybean accessions with reduced palmitic acid are desirable. However, for each year the bi-alleles of Map-6064 locus were observed not to be correlated with palmitic acid variation in our 421 worldwide soybean accessions (*p* > 0.05) (Fig. 5c). Nevertheless, considering the subpopulations individually, significant differences for palmitic acid (*p* < 0.05) were detected between the bi-alleles of Map-6064 locus (Additional file 3), suggesting that this locus was affected by population structure. Soybean accessions with Map-6064-C allele tended to contain higher levels of palmitic acid than those with Map-6064-T. Similar frequencies of favored allele were detected in landraces (25.2 %) and modern varieties (19.7 %) suggesting that no significant selection occurred in the Map-6064 locus during soybean genetic improvement. The allele frequencies of favorable Map-6064-T were further estimated for 117 NER, 48 NR and 180 SR accessions (Fig. 5d) and a large change was detected across subpopulations: the majority of NR accessions (77.1 %) possessed the Map-6064-T allele, whereas only a small proportion of NER and SR accessions (25.4 % and 8.3 % respectively) exhibited this favorable allele. Of 48 NR accessions examined, all modern varieties (3) and 34 of 45 landraces with Map-6064-T provide potentially important genetic resources for reducing palmitic acid in soybean seeds.

#### Stearic acid

Four of five loci significantly associated with stearic acid were near or overlapped with previously identified QTLs. The exception was Map-6568 on Gm15 (Table 2). Notably, the two most significant associations (Map-6506, *p* = 4.0E-08 and BARC-013927-01275, *p* = 1.4E-07) on Gm14 overlapped with the *fas* locus with respect to Satt474, Sat_189 and Satt556 [32, 49]. Map-6506 and BARC-013927-01275 explained 8.5 % and 7.8 % of stearic acid, respectively. Map-6506 is a synonymous mutation within exon 1 of *Glyma14g27990* (*SACPD-C* or *GmFAB2C*), a gene predicted to be involved in converting stearic acid into oleic acid. Besides Map-6506, two SNPs within *SACPD-C* - one non-synonymous and one nonsense mutation - had previously been found to cause variation of stearic acid levels in soybean [47, 50]. The second most significant SNP, non-synonymous BARC-013927-01275 in *Glyma14g27380* is within 752-kbp upstream of Map-6064, and exhibited a strong LD relationship with r^2^ of 0.91 and D’ of 0.99.

Unlike palmitic acid, a high level of stearic acid is desirable since it is not related to negative effects on human health and also offers potential for improving soybean oil quality in food-processing applications [51]. Significant differences in stearic acid were detected between bi-alleles at the Map-6506 locus across all three years studied (Fig. 5e). Average stearic acid of the accessions with favorable Map-6506-A allele were 3.86 %, 0.16 % higher than that of accessions with Map-6506-G (3.70 %). Desirable Map-6506-A represented only 27.3 % of accessions in the current diversity panel, and also in its three subpopulations (Fig. 5f).

### Estimation of the function of genes exhibiting significant marker-trait associations

In the current study, 32 annotated genes were identified to be significantly associated with one or two fatty acid components. Their function were estimated using comprehensive gene expression profiles of the RNA Seq-Atlas [52] from Soybase database (http://www.soybase.org/soyseq/). The expression level of all of 32 genes were compared among 14 tissues, including six tissues without seeds (root, nodule, young leaf, flower, pod shell-10 days after flowering DAF) and eight tissues with seeds (small pod, seeds-10DAF, −14DAF, −21DAF, −25DAF, −28DAF, −35DAF, and -42DAF) (Additional file 4). Five genes (including *Glyma01g24850*, *Glyma08g13060*, *Glyma15g17090*, *Glyma18g05980*, and *Glyma18g34290*) were not expressed in any tissue analyzed. Of the remaining 27 genes which were expressed in more than one tissue, three genes, *Glyma05g07630* with Map-0751, *Glyma05g34030* with Map-6077, and *Glyma14g27990* with Map-6506, exhibited preferential gene expression in tissues with seeds and the gene expression level of the later two increased with the seed development (Additional file 4). Seeds-specific *Glyma05g07630* was located nearby *GmFATB1a* [47] and identified to be significantly (*p* < 0.001) related with palmitic acid inferred from the association mapping. In *Arabidopsis thaliana*, *FatB* encodes a palmitoyl thioesterase, which is primarily involved in regulating the production of palmitic acid by catalyzing the conversion of 16:0-acyl carrier protein (ACP) (palmitic acid with ACP) to 18:0-ACP (stearic acid with ACP) [27]. In addition, it was reported that *Glyma05g34030* (*GmMFT*) may be a negative regulator of seed germination [53]. In this study, *GmMFT* maybe play another role on regulating linolenic acid in soybean seeds. Therefore, the functions of *Glyma05g34030* still need to be further analyzed through functional studies such as genetic transformation.

*Glyma14g27990* (*SACPD-C*) was one of four soybean isoforms of *SACPD* (also referred to as *FAB2*), a soluble desaturase which determines the relative proportions of saturated stearic acid and three unsaturated FAs [54]. The other three isoforms in soybean identified by comparative analyses were *GmSACPD-A* (*Glyma07g32850*) and *SACPD-B* (*Glyma02g15600*) and a pseudogene *SACPD-D* (*Glyma13g08990*) [45, 55]. It has been reported that both of *SACPD-B* and *SACPD-C* are responsible for the variation of seed stearic acid content in soybean [45, 47, 55]. However, in this study Map-6506 in *GmSACPD-C* was significantly (*p* < 0.001) associated with stearic acid, whereas Map-6017 in *GmSACPD-B* was significantly (*p* < 0.001) associated with linolenic acid. This suggests that the roles of the two orthologs of *GmSACPD* (*GmSACPD-B* and *GmSACPD-C*) are different (Fig. 3).

### Pleiotropic effects

Starting from saturated stearic acid, three unsaturated fatty acids, including oleic, linoleic and linolenic are synthesized as a result of the fatty acid desaturation pathway. Consequently, strong phenotypic correlations due to pleiotropy are expected for these four FA traits [5]. In accordance with this expectation we observed in our study that five out of the six pair-wise comparisons among these four FA traits were significantly (*p* < 0.01) correlated, except linolenic vs stearic acid. Moreover, association mapping analysis identified four SNPs (Map-6135, −6325, −6520 and −3670) exhibiting pleiotropic effects on stearic, oleic, linoleic or linolenic acid (Table 2). For example, as the precursor of linoleic acid, oleic acid exhibited a significant negative correlation with linoleic acid (Fig. 3). Map-6135 and Map-6325 were found to be significantly associated with both oleic and linoleic acid, but showed significant opposite effects, a decrease of oleic acid and an increase of linoleic acid with the Map-6135-A and Map-6325-C. These findings indicate that the QTNs exhibit strong pleiotropic effects.

## Conclusions

Due to predominant self-pollination, intensive selection during domestication and following genetic improvement, and genetic drift, extensive LD was observed for FA traits in soybean, especially in cultivated soybean (i.e. landrace and modern accessions) [23, 29, 56, 57]. Therefore, although resolution was limited, the association mapping approach employing diverse soybean cultivars allowed the identification of QTNs using a relatively small density of markers. In the present study, marker-trait association analysis detected 33 SNPs associated with at least one FA trait. Although further validations in independent panels or bi-parental populations are desirable, the co-localization of a group of associated loci with known genes/QTLs (as evidenced for Map-6017 in *GmSACPD-B,* Map-6506 in *GmSACPD-C,* and Map-6064 near *GmFATB1a* related with linolenic acid, stearic acid, and palmitic acid, respectively) suggests that the LD-based association mapping approach is suitable for detecting reliable associations with FA traits. These functional SNPs are essential tools for molecular soybean breeding programmes aimed at improving the FA quality of seeds and processed oil. In the future, labor/time-saving and cost effective SNP assays, such as Kompetitive Allele Specific PCR (KASP) and Cleaved Amplified Polymorphic Sequence (CAPS) assays, might be exploited based on these functional SNPs for assisted selection of specific desirable FA compositions, such as soybean varieties with increased stearic acid (>20 %) for food and industrial products [58]. It is important to note that some associations may have failed to be detected in this study owing to the limited marker density. A high density marker or sequencing-based analysis (i.e. re-sequencing, Genotyping by Sequencing etc.) should be conducted to further deepen our understanding of the genetic architecture of FAs in soybean using LD-based association mapping approach.

### Availability of data and materials

Four additional files in machine-readable format were uploaded in BMC Genomic website for supporting the results and findings found in this study, including

## Additional files

Additional file 1:
**Information for the 421 worldwide soybean accessions analyzed.** (XLS 106 kb) Additional file 2:
**Description of population genetic statistics of 1205 SNP markers in 421 soybean accessions.** (XLSX 130 kb) Additional file 3:
**Palmitic acid bi-alleles of Map-6064 in three subpopulations.** (TIFF 1235 kb) Additional file 4:
**The normalized transcription counts of 32 annotate genes with significant association signals.** Fourteen tissues including tissues without seeds (root, nodule, young_leaf, flower, pod shell-10DAF (Days After Flowering) and pod shell-14DAF) and tissues with seeds (seeds-14DAF,-21DAF, −25DAF, −28DAF, −35DAF, −42DAF and one cm pod) were displayed in order. RNA Seq data was from Soybase database (http://www.soybase.org/soyseq/) [52]. (TIFF 927 kb)
