# Supplementary figures and images for: Targeted association mapping demonstrating the complex molecular genetics of fatty acid formation in soybean
Source: BMC Genomics. 2015 Oct 23;16:841. doi: 10.1186/s12864-015-2049-4 (PMC4619020; doi:10.1186/s12864-015-2049-4)

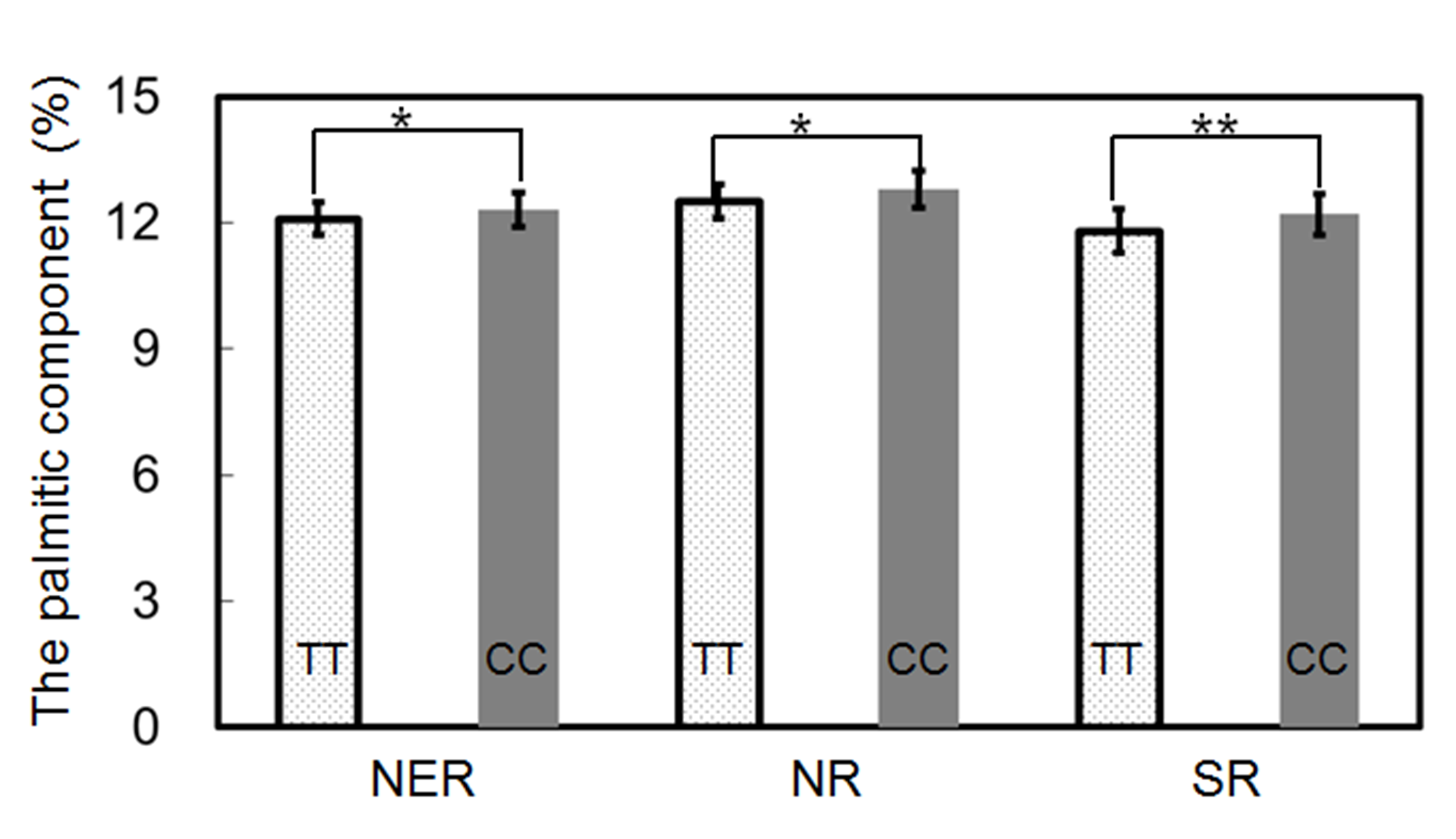

Supplement: Additional file 3: — Palmitic acid bi-alleles of Map-6064 in three subpopulations. (TIFF 1235 kb) [file 12864_2015_2049_MOESM3_ESM.tif]

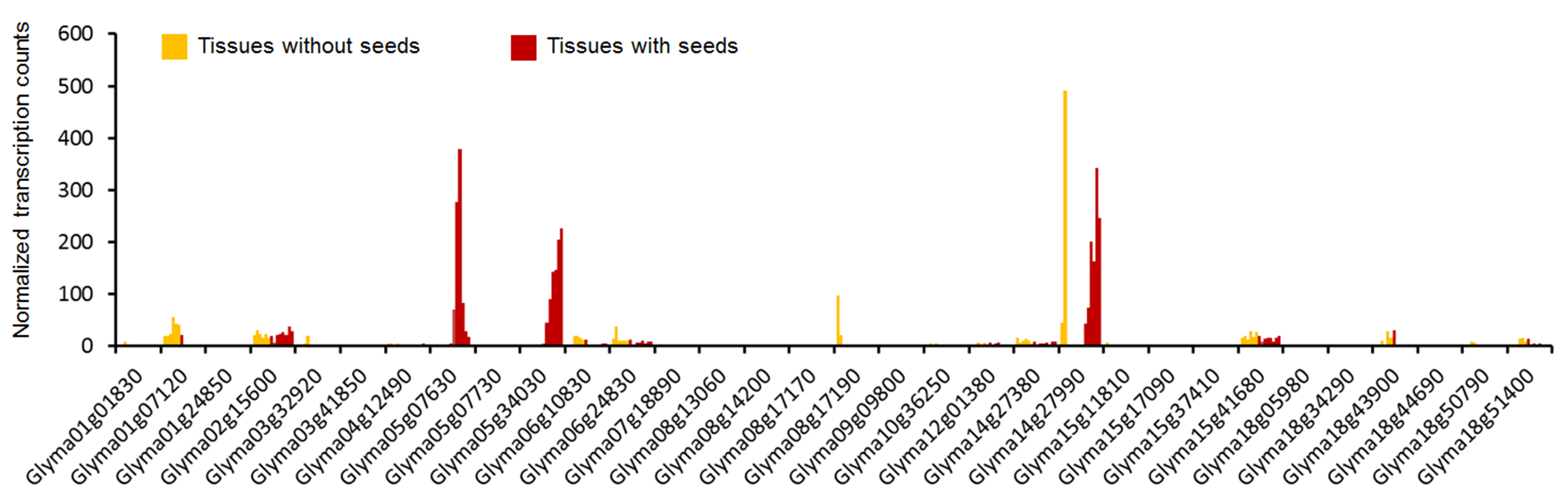

Supplement: Additional file 4: — The normalized transcription counts of 32 annotate genes with significant association signals. Fourteen tissues including tissues without seeds (root, nodule, young_leaf, flower, pod shell-10DAF (Days After Flowering) and pod shell-14DAF) and tissues with seeds (seeds-14DAF,-21DAF, −25DAF, −28DAF, −35DAF, −42DAF and one cm pod) were displayed in order. RNA Seq data was from Soybase database (http://www.soybase.org/soyseq/) [52]. (TIFF 927 kb) [file 12864_2015_2049_MOESM4_ESM.tif]
